# Comprehensive Analysis of Tumor Microenvironment Identified Prognostic Immune-Related Gene Signature in Ovarian Cancer

**DOI:** 10.3389/fgene.2021.616073

**Published:** 2021-02-10

**Authors:** Na Li, Biao Li, Xianquan Zhan

**Affiliations:** ^1^Science and Technology Innovation Center, Shandong First Medical University, Jinan, China; ^2^Key Laboratory of Cancer Proteomics of Chinese Ministry of Health, Xiangya Hospital, Central South University, Changsha, China; ^3^State Local Joint Engineering Laboratory for Anticancer Drugs, Xiangya Hospital, Central South University, Changsha, China; ^4^Department of Oncology, Xiangya Hospital, Central South University, Changsha, China; ^5^National Clinical Research Center for Geriatric Disorders, Xiangya Hospital, Central South University, Changsha, China

**Keywords:** ovarian cancer, immune-related-gene-signature, clinical characteristics, distribution of immune cells, distribution of tumor mutation burden

## Abstract

**Background:**

Accumulating evidence demonstrated that tumor microenvironmental cells played important roles in predicting clinical outcomes and therapeutic efficacy. We aimed to develop a reliable immune-related gene signature for predicting the prognosis of ovarian cancer (OC).

**Methods:**

Single sample gene-set enrichment analysis (ssGSEA) of immune gene-sets was used to quantify the relative abundance of immune cell infiltration and develop high- and low-abundance immune subtypes of 308 OC samples. The presence of infiltrating stromal/immune cells in OC tissues was calculated as an estimate score. We estimated the correlation coefficients among the immune subtype, clinicopathological feature, immune score, distribution of immune cells, and tumor mutation burden (TMB). The differentially expressed immune-related genes between high- and low-abundance immune subtypes were further used to construct a gene signature of a prognostic model in OC with lasso regression analysis.

**Results:**

The ssGSEA analysis divided OC samples into high- and low-abundance immune subtypes based on the abundance of immune cell infiltration, which was significantly related to the estimate score and clinical characteristics. The distribution of immune cells was also significantly different between high- and low-abundance immune subtypes. The correlation analysis showed the close relationship between TMB and the estimate score. The differentially expressed immune-related genes between high- and low-abundance immune subtypes were enriched in multiple immune-related pathways. Some immune checkpoints (PDL1, PD1, and CTLA-4) were overexpressed in the high-abundance immune subtype. Furthermore, the five-immune-related-gene-signature prognostic model (CCL18, CXCL13, HLA-DOB, HLA-DPB2, and TNFRSF17)-based high-risk and low-risk groups were significantly related to OC overall survival.

**Conclusion:**

Immune-related genes were the promising predictors of prognosis and survival, and the comprehensive landscape of tumor microenvironmental cells of OC has potential for therapeutic schedule monitoring.

## Introduction

Ovarian cancer (OC) is one of the most common gynecological tumors worldwide (Lheureux et al., 2019). The OC pathogenesis is concealed without specific symptoms in the early stage. Many patients are detected when the tumor has spread within the pelvis or belly (Kemppainen et al., 2019). In the advanced stage, OC becomes more difficult to be treated and could be fatal. More than half of late-stage OC patients treated by conventional oncology would not respond very well, which means that the current standardized protocols (surgery and chemotherapy) are not enough to combat this (Corrado et al., 2019). The survival rate of OC has been improved with the development of medicinal modes, but long-term survival rates are still poor (Giampaolino et al., 2019). Thus, it is necessary to identify the patient subset with worse survival who will need additional clinical therapy; for example, new targeted treatments, including poly(adenosine diphosphate-ribose) polymerase inhibitors, antiangiogenic drugs, and immune checkpoint inhibitors, potentially affect the improvement of survival (Elsherif et al., 2019). It is therefore necessary to establish new biomarkers that are related to cancer prognosis and survival, and a complete biological database benefits the construction of a more common prognostic signature for OC.

Accumulating evidence demonstrated that the immune system played an important role in cancer initiation, progression, and therapeutic responses (Abbott and Ustoyev, 2019). OC was one of the immunogenic tumors that responded very well through targeting the immune checkpoints (Ghisoni et al., 2019). When a single-agent antibody was used to inhibit the cytotoxic T lymphocyte-associated protein 4 (CTLA-4) or PD-L1 axis, encouraging results were obtained with a median response rate of 10–15% in OCs (Hamanishi et al., 2015). Interestingly, the combination of anti-CTLA4 ipilimumab and anti-PD1 nivolumab showed an overall response rate (ORR; 34%), which almost doubled the effect of nivolumab monotherapy (Mills and Fuh, 2017). The previous studies preliminarily reported the prognostic value of the immune system in OC (Odunsi, 2017) and revealed the significance of tumor-related signaling pathways in the tumor microenvironment (Newsted et al., 2019). For example, immune cell types in the tumor microenvironment, such as tumor-associated macrophages, were significantly different in OC patients with chemoresistance (An and Yang, 2020). M1 macrophages were significantly associated with better outcomes among the high-grade and late-stage OC patients (HR: 0.77–0.83). Neutrophils were significantly associated with worse outcomes among the high-grade and late-stage OC patients (HR: 1.14–1.73) (Gao et al., 2020). In OC patients, inflammatory cytokines also influenced the outcome of patients. For example, IL-22 and TNF-á were increased in OC patients with stages III–IV compared to stages I–II (Wang et al., 2017). BRCA1 or BRCA2 mutations were very common in the OC patients, which were closely related to the prognosis of OC patients (Birkbak et al., 2013). One study found that the elevated tumor mutation burden (TMB) was significantly associated with the efficacy of immunotherapy, which prolonged clinical response to the anti-PD-L1 antibody in platinum-resistant recurrent OC (Morse et al., 2017). More importantly, the abundance of immune cells and other stromal cells in the tumor microenvironment can be estimated and scored with multiple computational methods (Becht et al., 2016). These methodologies have been used to investigate the relationship between the immune system and OC prognosis (Liu et al., 2020), and some mechanisms that immune-related genes were involved in within the immunotherapy response have been identified in OCs (Shen et al., 2019). However, the comprehensive landscape and immune-related gene signature have not been elucidated yet.

In this study, 308 OC cases from TCGA database were divided into two immune subtypes—high and low abundance groups of immune cell infiltration based on the mixed cellular gene expression data. These two immune subtypes were systematically correlated with clinical characteristics, ESTIMATE results (including stromal score, immune score, and estimate score), and mutation information. These correlation results were used to predict and evaluate the clinical outcomes of OC. Further, lasso regression was used for the identification of the five-immune-related-gene signature model (CCL18, CXCL13, HLA-DOB, HLA-DPB2, and TNFRSF17) to improve the predictive accuracy for OC overall survival. This study aimed to quantify the cellular compositions of the immune response and explore its association with the OC prognosis. The immune-based prognostic signature will provide potential value for prognostic prediction and tailored immunotherapy of OC.

## Materials and Methods

### OC Cohort Acquisition

The OC RNA-sequencing data (FPKM values), mutation data, and clinical follow-up data were obtained from the public database TCGA^1^. Patients with complete follow-up information and survival status were selected to match with their RNA-seq data. The main outcome of our study was overall survival. Patients without survival information were removed for further evaluation. Following these criteria, 308 OC cases were involved in this study (Supplementary Tables 1–3). Data were analyzed with the R (version 3.4.0) and R Bioconductor packages.

Also, OC gene expression datasets were systematically searched that were publicly available with full clinical annotations. The data from GSE13876 “Survival Related Profile, Pathways and Transcription Factors in Ovarian Cancer” were downloaded to validate the reliability of the built model. GSE13876 developed a gene expression profile associated with overall survival in advanced-stage serous ovarian cancer (*n* = 415). The raw data from the microarray datasets generated by Affymetrix and Illumina were downloaded from the Gene Expression Omnibus2, including gene expression data, and survival information.

### Quantification of the Abundance of Immune Cell Infiltration in the Tumor Microenvironment

To estimate the population-specific immune infiltration, the single-sample gene-set enrichment analysis (ssGSEA) was used to quantify the abundance of tumor microenvironment cell infiltration in each sample based on the gene expression data (Wu et al., 2016). Briefly, the ssGSEA applied gene signatures expressed by immune cell populations to individual cancer samples. The computational approach included immune cell types that are involved in innate immunity and adaptive immunity. Tumors with qualitatively different immune cell infiltration patterns were grouped with hierarchical agglomerative clustering based on Euclidean distance and Ward’s linkage. In total, 24 human tumor microenvironment cell subtypes were evaluated, including M0 macrophages, M1 macrophages, M2 macrophages, memory B cells, naive B cells, endothelial cells, eosinophils, activated dendritic cells, resting dendritic cells, fibroblasts, activated NK cells, resting NK cells, activated mast cells, resting mast cells, monocytes, resting CD4 memory T cells, naive CD4 T cells, neutrophils, plasma cells, follicular helper T cells, gamma delta T cells, activated CD4 memory T cells, CD8 T cells, and regulatory T cells (Supplementary Table 4). The ssGSEA analysis was conducted with GSVA R package (version: 3.12)^2^.

### Sparse Hierarchical Clustering for OC Samples

Tumors with different qualitative microenvironment cell infiltration patterns were grouped with sparse hierarchical clustering based on Euclidean distance and Ward’s linkage. The sparse k-means method was used to establish the optimal number of tumor groups. This method used the genes in each node and metanode. Briefly, classification consistency was tested with random forest. The sparse hierarchical clustering algorithm was used to analyze the data that contained the variables selected by the sparse K-means method, which optimized the classification of samples into two subtypes (Supplementary Table 5). This procedure was performed with the Sparcl R package (version 1.0.3) (Prado-Vázquez et al., 2019) and was repeated 1,000 times to ensure the stability of classification^3^.

### Estimation of Infiltrating Cells and Tumor Purity in OC

ESTIMATE (Estimation of Stromal and Immune cells in MAlignant Tumor tissues using Expression data) is a tool to predict tumor purity and the presence of infiltrating stromal/immune cells in tumor tissues with gene expression data (Supplementary Table 6). The ESTIMATE algorithm is based on single sample Gene Set Enrichment Analysis and generates three scores: ImmuneScore (that represents the infiltration of immune cells in tumor tissue), StromalScore (that captures the presence of stroma in tumor tissue), and ESTIMATEScore (that infers tumor purity). They all positively correlated with the ratio of immune, stroma, and the sum of both, respectively, which means the higher the respective score, the larger the ratio of the corresponding component in microenvironment cell (Yoshihara et al., 2013). Estimations of infiltrating cells and tumor purity in OC were analyzed by Estimate R package (version 3.5.1) using the ESTIMATE algorithm^4^.

### The Proportions of Immune Cells in OC

The CIBERSORT algorithm and the LM22 gene signature were used to quantify the proportions of immune cells in the OC samples, which allows for highly sensitive and specific discrimination of 22 human immune cell phenotypes. CIBERSORT was a deconvolution algorithm that uses a set of reference gene expression values (a signature with 547 genes) considered a minimal representation for each cell type and, based on those values, infers cell type proportions in data from bulk tumor samples with mixed cell types using support vector regression. Gene expression profiles were prepared with standard annotation files, and data were uploaded to the CIBERSORT web portal^5^, with the algorithm run using the LM22 signature and 1,000 permutations (Supplementary Table 7). The correlation analysis between immune cells was conducted with Corrplot R package (version 0.84) based on Pearson correlation analysis (Supplementary Table 8).

### The Distribution of Tumor Mutation Burden (TMB) and TMB Score

TMB was calculated as mutations per megabase (mut/Mb). Mutational signature analysis was performed for tumors with somatic mutation counts of at least 10. With the development of cancer genomics, mutation annotation format (MAF) was widely accepted and used to store the detected somatic variants. Maftools is an R package that is published in Bioconductor^6^, and is specially used to visualizethe information in MAF files. The distribution of tumor mutation burden (TMB) was conducted by Maftools R package (Supplementary Table 3), which generated four results, including the summary of TMB, waterfall of mutation genes, interaction of mutation genes, and TMB score (Supplementary Table 9). Furthermore, the association between the expression of top mutation genes and drug sensitivity was performed by Corrplot R package (version 0.84) with the Pearson correlation coefficient (*r* > 0.5, and *p* < 0.05) based on the corresponding data from CellMiner^7^.

### Differentially Expressed Genes (DEGs) Associated With the Immune Subtypes

To identify genes associated with the immune subtype, OC patients were grouped into high- and low-abundance groups of immune cell infiltration (Immunity-H; Immunity-L). DEGs between these two groups were determined with the R package limma package (Bioconductor version 3.0), which implements an empirical Bayesian approach to estimate gene expression changes using moderated *t*-tests (Supplementary Table 10). DEGs among immune subtypes were determined with significance criteria (adjusted *P*-value < 0.05). The adjusted *P*-value for multiple testing was calculated with the Benjamini-Hochberg correction. The immunology database and analysis portal (IMMPORT) website^8^ is funded by the NIH, NIAID, and DAIT in support of the NIH mission to share data with the public. The expression information of immune-related genes of each sample was extracted with ImmPort, and differentially expressed immune-related genes (DEIRGs) were identified, including genes that were directly or indirectly involved in immune responses (Supplementary Tables 11,12).

### Functional and Pathway Enrichment Analysis

Gene-annotation enrichment analysis of DEGs was performed with the clusterProfiler R package (version 3.0.4)^9^. Gene Ontology (GO) terms (Supplementary Table 13) and Kyoto Encyclopedia of Genes and Genomes (KEGG) pathways (Supplementary Table 14) were identified with a strict cutoff value *P* < 0.01 and a false discovery rate (FDR) < 0.05. The upregulated and downregulated pathways were also identified between two immune subtypes with a gene set enrichment analysis (GSEA) of the adjusted expression data for all transcripts. Gene sets were downloaded from the MSigDB database of Broad Institute^10^. Enrichment *P*-values were based on 10,000 permutations and subsequently adjusted with the Benjamini-Hochberg multiple testing to control the FDR. All DEIRGs were mapped in the protein–protein interaction (PPI) network in the STRING^11^ database (Supplementary Table 15) to evaluate their interactive associations. Subsequently, the PPIs were analyzed with Cytoscape software (version 3.2.1; National Resource for Network Biology) to obtain hub modules with the molecular complex detection (MCODE) (score > 7).

### Lasso Regression for OC Tissues

The OC samples were divided into two groups according to the mean value of DEIRG expressions. Overall survival was calculated with the Kaplan-Meier method and compared to the log-rank test. The *p* < 0.05 was considered a statistical significance. Further, the overall survival-related DEIRGs were selected to construct lasso regression that examines the relationship between gene signatures and OC risk score. Lasso regression was constructed by the glmnet R package^12^. The risk scores of OC samples were calculated according to the expression of the selected overall survival-related DEIRGs and were stratified into high- and low-risk score groups based on the median value of the risk scores. The Kaplan-Meier method was used to evaluate the availability of the prognostic model. In addition, the association of clinical characteristics with overall survival was analyzed in OC patients with the univariate and multivariate Cox regression model.

### Statistical Analysis

For between-group comparisons, statistical significance was estimated with unpaired Student’s *t*-tests for normally distributed variables and with Mann-Whitney *U*-tests (also called the Wilcoxon rank-sum test) for non-normally distributed variables. The Spearman correlation coefficient was calculated between the TMB score and ESTIMATEScore by Graphpad prism 8. In all cases, *p* < 0.05 was considered as statistical significance. Benjamini-Hochberg multiple testing and FDR were calculated to correct the *p*-value in DEGs, GO, and KEGG analysis. The Kaplan-Meier method was used to generate survival curves for the subgroups in each data set, and the Log-rank (Mantel-Cox) test was used to determine the statistical significance of differences. The hazard ratios for univariate analyses were calculated with a univariate Cox proportional hazard regression model.

## Results

### Sparse Hierarchical Clustering Showed Significant Association With Clinical Features and ESTIMATE Algorithm

**FIGURE 1:**
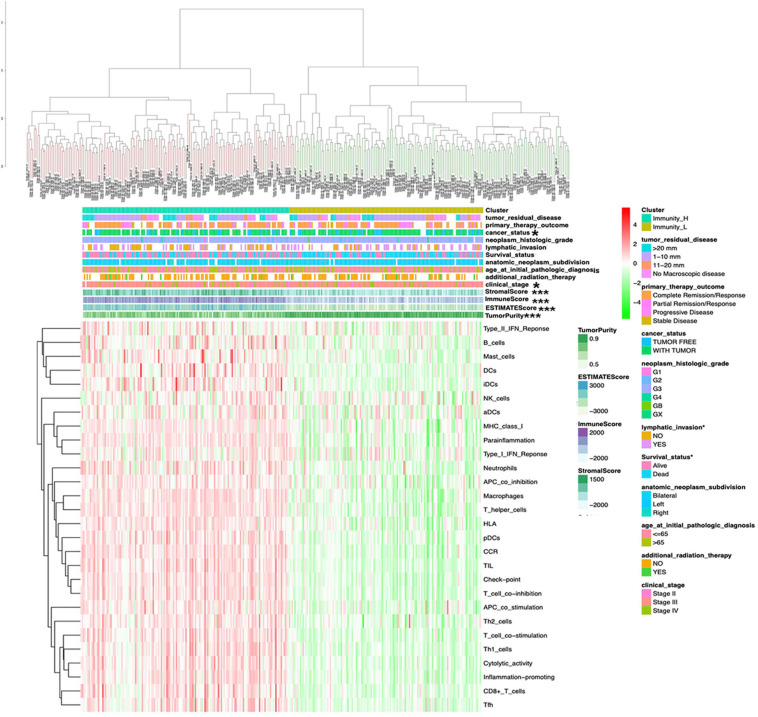
The clinical and ESTIMATE algorithm association of immune clusters. The heatmap shows the OC samples were divided into two groups (immune relative high abundance of immune cells infiltration group and low abundance of immune cells infiltration group). The distribution of clinical features was compared between the high- and low-abundance immune groups. The association of ESTIMATE algorithm (including immunescore, stromalscore, and ESTIMATEscore) was compared between high- and low-risk immune groups. *p < 0.05, ***p < 0.001.

The OCcohort included RNA-sequencing data from a total of 308 patients (Supplementary Table 1) with complete clinical follow-up information, including survival status, additional radiation therapy, age at initial pathological diagnosis, anatomic neoplasm subdivision, clinical stage, lymphatic invasion, histologic grade, cancer status, primary therapy outcome, and tumor residual disease (Supplementary Table 2). The abundance of each tumor microenvironment cell infiltration based on the gene expression data in OC was calculated by ssGSEA (Supplementary Table 4), including the immune cell information (aDCs, APC co inhibition, APC co stimulation, B cells, CCR, CD8^+^ T cells, checkpoint, cytolytic activity, DCs, HLA, iDCs, inflammation-promoting, macrophages, mast cells, MHC class I, neutrophils, NK cells, parainflammation, pDCs, T cell co-inhibition, T cell co-stimulation, T helper cells, Tfh, Th1 cells, Th2 cells, TIL, type I IFN response, and type II IFN response). The OC samples provided an optimum classification into two subtypes with the sparse hierarchical clustering algorithm based on the data containing immune cell information, which named high- and low-immunity abundance groups of the immune cell infiltration (Immunity-H: *n* = 156; Immunity-L, *n* = 152) (Supplementary Table 5). The ssGSEA-bsed ESTIMATE algorithm generated three scores: the immune score, stromal score, and ESTIMATE score (Supplementary Table 6). The correlations between clinical features and tumor microenvironment cell infiltration were tested with the chi-square test based on those two immune subtypes. The sample clusters were significantly related to those clinical characteristics, including clinical stages (II, III, and IV) and cancer status (with cancer or without cancer). The sample clusters were also significantly positively related to immune score, stromal score, and ESTIMATE score; and were negatively related to tumor purity (Figure 1).

### The Distribution of Immune Cells in Different Immune Subtypes and Correlation With Clinical Features and Survival

**FIGURE 2:**
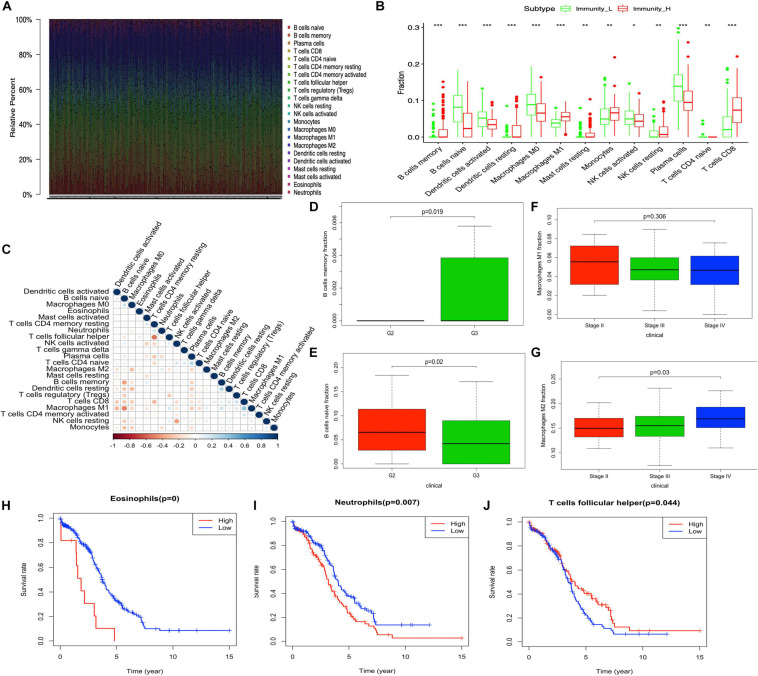
The distribution of immune cells between high- and low-abundance immune groups and corresponding clinical features. **(A)** Barplot showing the proportion of 22 kinds of immune cells in OC samples. Column names of the plot are the sample ID. **(B)** Boxplot shows the ratio differentiation of 13 kinds of immune cells between high- and low-abundance immune groups, and the Wilcoxon rank sum was used for the significance test. **(C)** Heatmap showing the correlation between 22 kinds of immune cells and numeric in each tiny box indicating the *p*-value of the correlation between two kinds of cells. The shade of each tiny color box represents the corresponding correlation value between two cells, and the Pearson coefficient was used for significance test. **(D,E)** The correlation of B cells memory and B cells naïve with clinicopathological grade characteristics. Wilcoxon rank sum or Kruskal–Wallis rank sum test served as the statistical significance test. **(F,G)** The correlation of M1 macrophages and M2 macrophages with clinicopathological stage characteristics. Wilcoxon rank sum or Kruskal–Wallis rank sum test served as the statistical significance test. **(H–J)** Kaplan–Meier survival analysis for Eosinophils, neutrophils, and T cells follicular helper in OC. A comparison with the median determined immune cell group. *P*-value was verified by log-rank test. *p < 0.05, **p < 0.01, and ***p < 0.001.

The tumor-immune infiltration of the 308 OC samples was summarized (Figure 2A and Supplementary Table 7). The distribution of immune cells was significantly different between Immunity-H and Immunity-L groups, including B cells naïve, B cells memory, plasma cells, T cells CD8, T cells CD4 naïve, NK cells resting, NK cells activated, monocytes, macrophages M0, macrophages M1, dendritic cells resting, dendritic cells activated, and mast cells resting (Figure 2B). The tumor-immune infiltration cell heatmap depicted a comprehensive landscape of tumor-immune cell interactions and cell lineages in OC, which indicated that some immune cells were particularly closely connected to others, for example, T cells follicular helper and T cells CD4 memory resting, macrophages M2 and macrophages M0, B cells naïve and B cells memory, dendritic cells resting and eosinophils, macrophages M1 and B cells naïve, and T cells CD8 and T cells CD4 memory resting (Figure 2C and Supplementary Table 8). In terms of clinical characteristics, B cells memory was increased in patients with grade 3 compared to grade 2 in OC (Figure 2D). B cells naïve was decreased in patients with grade 3 compared to grade 2 in OC (Figure 2E). M1 macrophages were present a significantly decreased trend among stages II, III, and IV (Figure 2F), while M2 macrophages were present a significantly increased trend among stages II, III, and IV (Figure 2G). Eosinophils, neutrophils, and T cells follicular helper were significantly related to OC survival (Figures 2H–J).

### Associations Between ESTIMATE Score and Mutation

**FIGURE 3:**
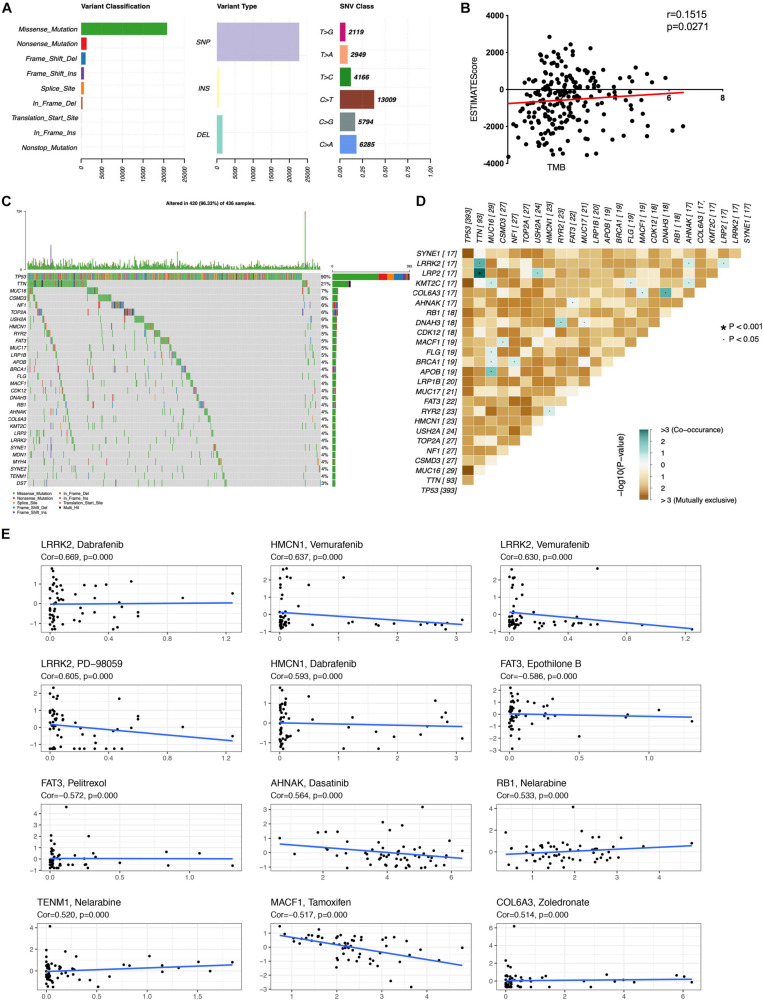
Associations of mutation in immune subtype. **(A)** The overall summary of mutation information in the OC cohort. **(B)** The correlation of TMB with ESTIMATEscore in OC. **(C)** The waterfall plot shows the top 30 mutated genes in OC cohort and their mutation information. **(D)** The significant co-occurrence of gene mutations in OC. **(E)** The associations of top mutation gene expressions with drug sensibility. TMB, Tumor mutation burden. The statistical significance was defined as *p* < 0.05.

The distribution of tumor mutation was plotted to explore the relationship between immune and mutation statuses of OC (Figures 3A,C and Supplementary Table 3). The mutation of the top 30 genes was plotted, including TP53, TNN, MUC16, CSMD3, NF1, TOP2A, USH2A, HMCN1, RYR2, FAT3, MUC17, LRP1B, APOB, BRCA1, FLG, MACF1, CDK12, DNAH3, RB1, AHNAK, COL6A3, KMT2C, LRP2, LRRK2, SYNE1, MDN1, MYH4, SYNE2, TENM1, and DST. Furthermore, TMB score was significantly associated with the ESTIMATE score, which indicated that TMB might be significantly associated with the efficacy of immunotherapy (Figure 3B and Supplementary Table 9). The significant co-occurrences of gene mutations were plotted (Figure 3D), such as LRP2 and TNN, TNN and LRRK2, MUC16 and KMT2C, MUC16 and BRCA1, MUC16 and APOB, NF1 and BRCA1, DNAH3 and COL6A3, and DNAH3 and RYR2. It was well revealed that tumors were dependent on driver mutations that promote and maintain the malignant phenotype. Moreover, the associations of top mutation gene expressions with drug sensibility were explored. Some mutation genes showed positive associations with drug sensibility, including COL6A3 and Zoledronate, TENM1 and Nelarabine, RB1 and Nelarabine, AHNAK and Dasatinib, HMCN1 and Dabrafenib, LRRK2 and PD-98059, LRRK2 and Vemurafenib, HMCN1 and Vemurafenib, and LRRK2 and Dabrafenib (Figure 3E). Some mutation genes showed negative associations with drug sensibility, including FAT3 and Epothilone B, FAT3 and Pelitrexol, and MACF1 and Tamoxifen (Figure 3E). Those drug sensibility-associated mutation genes were the potential drug therapeutic targets.

### GO and KEGG Analysis of DEGs Significantly Enriched in Immune System

**FIGURE 4:**
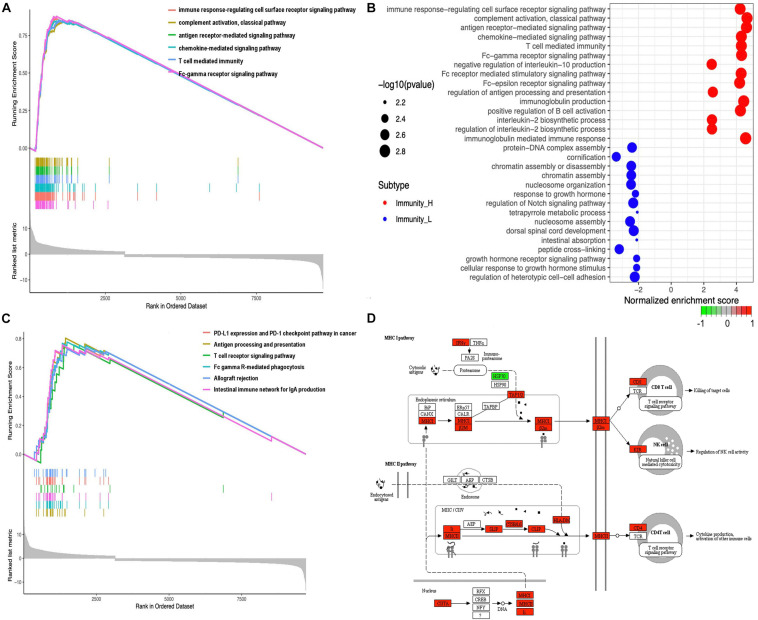
Functional and pathway enrichment analyses. **(A)** The GO enrichment analysis in different expressed genes between the high- and low-abundance immune groups. Only gene sets with NOM *p* < 0.05 and FDR *q* < 0.05 (adjusted *p*-value using the Benjamini-Hochberg procedure to control the FDR) were considered significant. Only six leading gene sets were displayed in the plot. **(B)** The up- and downregulated GO pathways among immune subtypes by running a gene set enrichment analysis (GSEA) in different expressed genes. Enrichment *P*-values were based on 10,000 permutations and subsequently adjusted for multiple testing using the Benjamini-Hochberg procedure to control the FDR. **(C)** The KEGG enrichment analysis in different expressed genes between the high- and low-abundance immune groups. Only gene sets with NOM *p* < 0.05 and FDR *q* < 0.05 (adjusted *p*-value using the Benjamini-Hochberg procedure to control the FDR) were considered significant. Only six leading gene sets were displayed in the plot. **(D)** MHC-I pathway as an example for enriched KEGG.

A total of 20,530 genes were analyzed with the limma package to identify the underlying biological characteristics of immune subtypes, and 9712 DEGs were acquired (Supplementary Table 10). Next, GO and KEGG enrichment analyses of DEGs revealed a total of 1,064 statistically significant GO enrichments (Supplementary Table 13) and 64 statistically significant KEGG enrichments (Supplementary Table 14). GO enrichment results found that DEGs were closely related to immune processes; for example, immune response-regulating cell surface receptor signaling pathway, complement activation pathway, antigen receptor-mediated signaling pathway, chemokine-mediated signaling pathway, T-cell-mediated immunity, Fc-gamma receptor signaling pathway, negative regulation of interleukin-10 production, Fc receptor-mediated stimulatory signaling pathway, Fc-epsilon receptor-signaling pathway, regulation of antigen processing and presentation, immunoglobulin production, positive regulation of B-cell activation, interleukin-2 biosynthetic process, regulation of interleukin-2 biosynthetic process, immunoglobulin mediated immune response, B-cell-mediated immunity, B-cell receptor signaling pathway, Fc receptor signaling pathway, and phagocytosis. Here, we showed the top six enrichments (Figure 4A) and top 15 enrichments that were upregulated and downregulated between two immune subtypes (Figure 4B) derived from GO analysis. KEGG enrichment results found that DEGs were closely related to immune processes too; for example, PD-L1 expression and the PD-1 checkpoint pathway in cancer, antigen processing and presentation, T-cell receptor signaling pathway, Fc gamma R-mediated phagocytosis, intestinal immune network for IgA production, B-cell receptor signaling pathway, primary immunodeficiency, Th17 cell differentiation, viral protein interaction with cytokine and cytokine receptor, NF-kappa B signaling pathway, TNF signaling pathway, Th1 and Th2 cell differentiation, and chemokine signaling pathway. Here, we showed the top six enrichments (Figure 4C) and the MHC-I pathway (Figure 4D) derived from KEGG analysis.

### IRG Expression Changes and Hub Molecules in Immune Subtype

**FIGURE 5:**
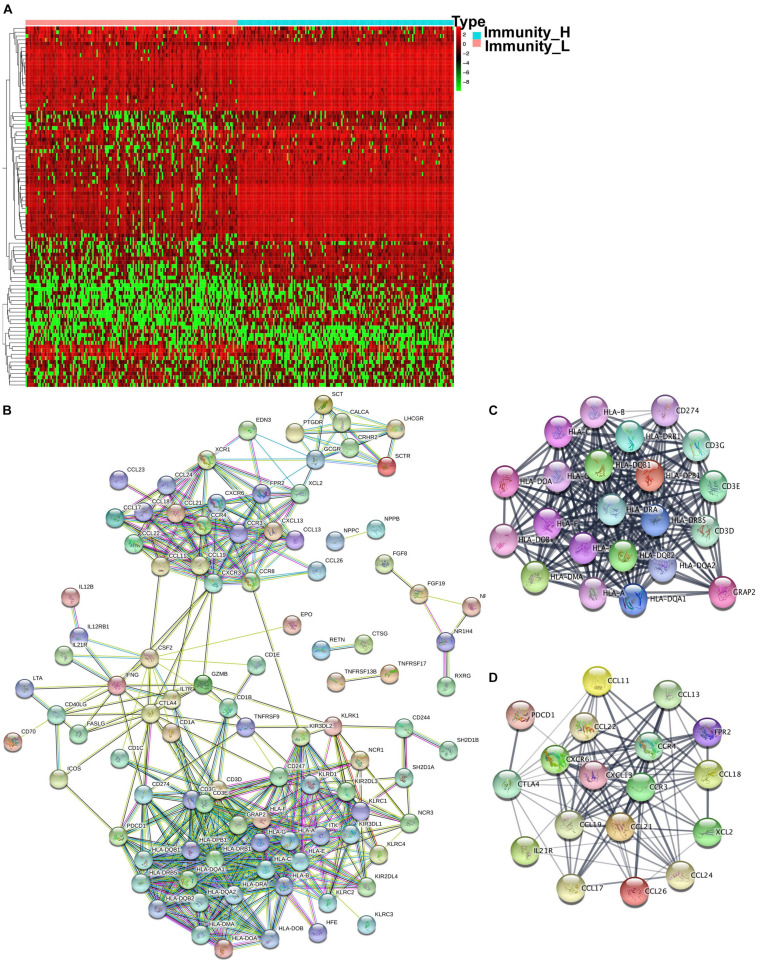
The different expressed immune-related genes and PPI network in OC tissues. **(A)** The heatmap of OC tissues and the different expressed immune-related genes between the high- and low-abundance immune groups. **(B)** PPI network of those different expressed immune-related genes. **(C,D)** Hub modules were analyzed using MCODE (module 1 score = 20.09 and module 2 score = 11.75).

The GO and KEGG analysis showed that DEGs were significantly enriched in the immune system, which prompted IRGs expression changes between immune subtypes. The expression information of IRGs was extracted from each sample according to the list of IRGs curated by the Immunology Database and Analysis Portal (IMMPORT) website, which included genes that were directly or indirectly involved in immune responses. A total of 998 IRGs were curated by the IMMPORT dataset in OC (Supplementary Table 11), among which around 118 were identified as DEIRGs between two immune subtypes (Figure 5A and Supplementary Table 12). Those DEIRGs were used to construct a PPI network (Figure 5B). The entire PPI network was analyzed with MCODE, and two hub modules (module 1 score = 20.09 and module 2 score = 11.75) were chosen (Figures 5C,D). Thus, a total of 147 hub molecules was identified according to hub modules. Hub module 1 contained 22 hub molecules, including HLA-A, HLA-B, HLA-C, HLA-DMA, HLA-DOA, HLA-DOB, HLA-DPB1, HLA-J, HLA-DQA1, HLA-DQA2, HLA-DQB1, HLA-G, HLA-DRA, HLA-DRB1, HLA-DRB5, HLA-DRB6, HLA-DPB2, CD274, CD3G, CD3E, CD3D, and GRAP2 (Figure 5C). Hub module 2 contained 18 hub molecules, including CCL26, PDCD1, CCL21, CCL22, CCL24, CCL17, FPR2, CCL18, CCL19, IL21R, CXCR6, CCL11, CCL13, XCL2, CCR3, CCR4, CXCL13, and CTLA4 (Figure 5D). Those hub molecules assisted in the understanding of the key molecules in the PPI network.

### HLA System and Immune Checkpoint in Immune Subtype

**FIGURE 6:**
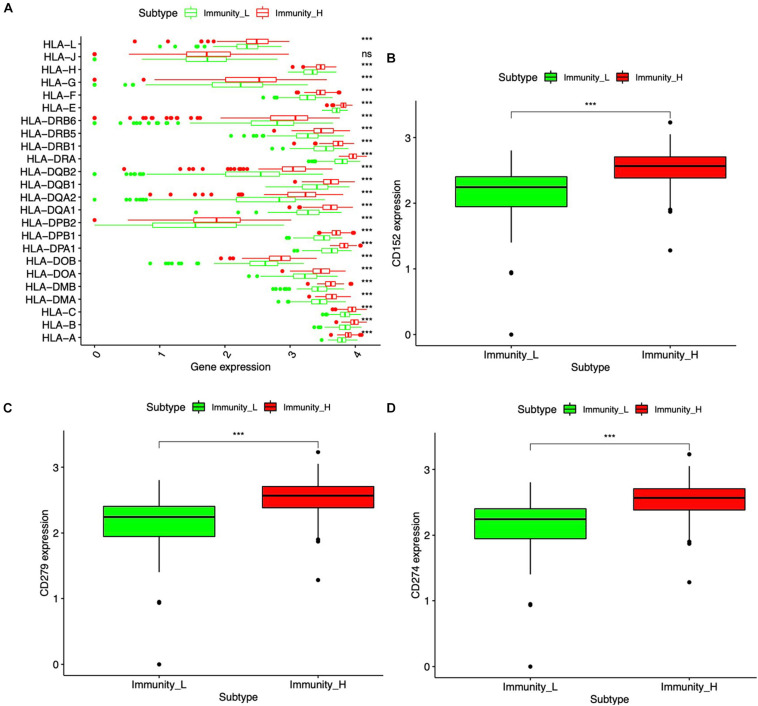
HLA system and immune checkpoint between the high- and low-abundance immune groups. **(A)** Boxplot of the difference between high- and low-abundance immune groups in the HLA family. **(B)** Boxplot of the difference in CTLA-4 (CD152) between high- and low-abundance immune groups. **(C)** Boxplot of the difference in PD-1 (CD279) between high- and low-abundance immune groups. **(D)** Boxplot of the difference in PD-L1 (CD274) between high- and low-abundance immune groups. Wilcoxon rank sum was used for the significance test. ***p < 0.001.

Major histocompatibility complex (MHC), a group of genes that code proteins, was found on the surfaces of cells that help the immune system recognize foreign substances. MHC proteins were found in all higher vertebrates. In human beings, the complex was also called the human leukocyte antigen (HLA) system. According to the hub molecules obtained from the PPI network, the HLA system and immune checkpoints (PD1, PDL1, and CTLA-4) were identified. The expression information of the HLA family and immune checkpoints from DEIRGs were shown (Figure 6), which indicated that HLA family and immune checkpoints were significantly different between Immunity-H and Immunity-L groups. Specifically, PD1, PDL1, and CTLA-4 were significantly highly expressed in the Immunity-H group. The HLA family, including HLA-A, HLA-B, HLA-C, HLA-DMA, HLA-DMB, HLA-DOA, HLA-DOB, HLA-DPA1, HLA-DPB1, HLA-DPB2, HLA-DQA1, HLA-DQA2, HLA-DQB1, HLA-DQB2, HLA-DRA, HLA-DRB1, HLA-DRB5, HLA-DRB6, HLA-E, HLA-F, HLA-G, HLA-H, and HLA-L, showed significant differences between Immunity-H and Immunity-L groups.

### Development of the Immune-Based Prognostic Signature for OC

**FIGURE 7:**
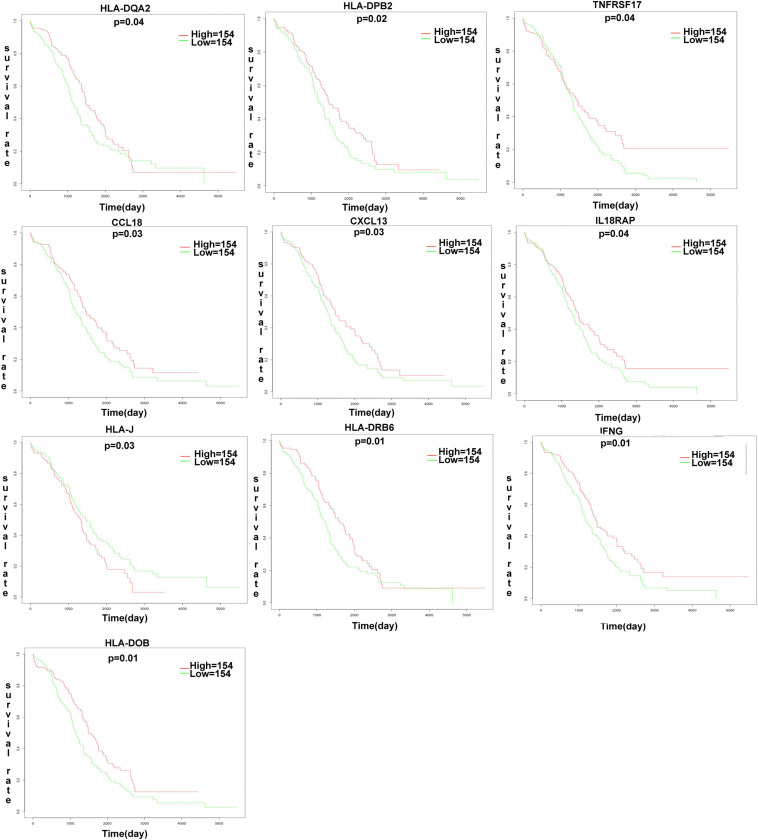
The K-M plot analysis revealed that 10 out of 118 DEIRGs were significantly associated with OC overall survival (*p* < 0.05), including CCL18, CXCL13, HLA-DOB, HLA-DPB2, HLA-DQA2, HLA-DRB6, HLA.J, IFNG, IL18RAP, TNFRSF17. *P*-value was verified by log-rank test.

**FIGURE 8:**
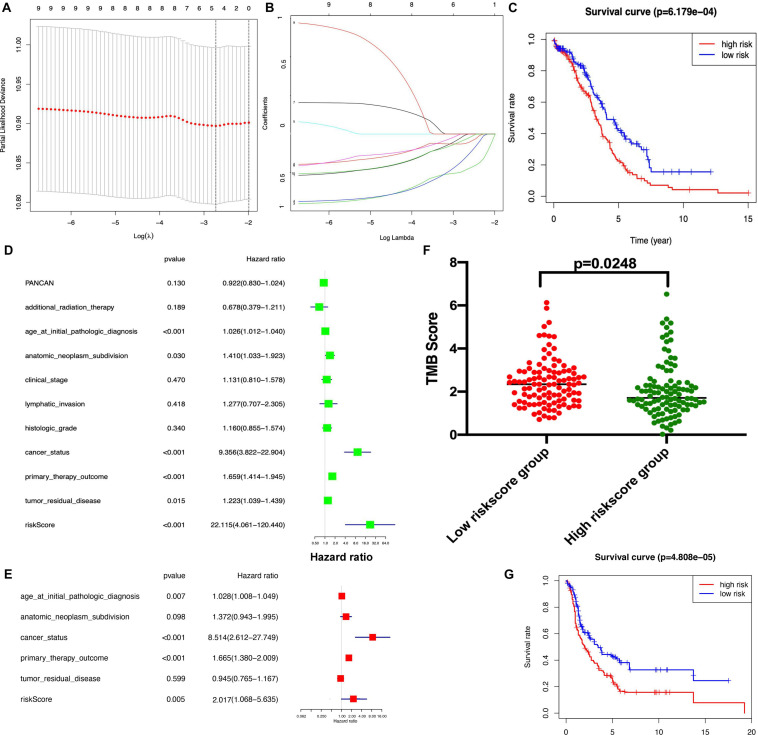
Lasso regression identified the prognostic model of five-immune related gene signature. **(A,B)**. Lasso regression complexity was controlled by lambda using the glmnet R package. **(C)** Overall survival analysis between high-risk score and low-risk score groups based on TCGA dataset. **(D)** The univariate analysis of risk factors in OC. **(E)** The multivariate analysis of risk factors in OC. **(F)** The association between TMB and risk score group. **(G)** Overall survival analysis between high-risk score and low-risk score groups based on GEO dataset. Statistical significance was set as *p* < 0.05.

The K-M plot analysis revealed that 10 out of 118 DEIRGs were significantly associated with OC overall survival (*p* < 0.05), including CCL18, CXCL13, HLA-DOB, HLA-DPB2, HLA-DQA2, HLA-DRB6, HLA-J, IFNG, IL18RAP, and TNFRSF17 (Figure 7). Most of them were hub molecules in the PPI network. Furthermore, lasso regression was performed to identify the five-immune-related gene signature model (CCL18, CXCL13, HLA-DOB, HLA-DPB2, and TNFRSF17) to improve the predicted accuracy for overall survival in OC, when log (lambda) was between −2 and −3 (Figures 8A,B). The survival risk score was calculated as (-0.10182 × expression level of CCL18) + (-0.2125 × expression level of CXCL13) + (-0.6473 × expression level of HLA-DOB) + (-0.5321 × expression level of HIBCH) + (-0.120 × expression level of HLA-DPB2) + (-0.2076 × expression level of TNFRSF17). The OC samples were divided into two groups (high-risk score group = 153, and low-risk score group = 154) according to the median value of risk score (risk score value = 0.639) (Supplementary Table 16). Additionally, overall survival showed statistical significance between high-risk and low-risk groups (Figure 8C). The five-immune-related gene signature was consistent with the single-factor analysis of genes using Cox regression. The univariate analysis revealed that age at initial pathological diagnosis, anatomic neoplasm subdivision, cancer status, primary therapy outcome, tumor residual disease, and risk score were significantly correlated with overall survival (Figure 8D). The univariate analysis revealed that age at initial pathological diagnosis, cancer status, primary therapy outcome, and risk score were significantly correlated with overall survival (Figure 8E). The risk score of the prognostic model in OC was negatively correlated with the TMB score (Figure 8F) (*p* = 0.0248).

### The Independent Verification by GEO Dataset

To validate the reliability of the established prognostic model based on the TCGA database, a testing dataset that was derived from 415 OC patients downloaded from the GEO dataset^13^, and these 405 patients were stratified into a low-risk score group (*n* = 207) and high-risk score group (*n* = 208) according to the median risk score based on the immune-based prognostic model. There was a significant difference in the OS rate between the two groups, and the OS rate was significantly lower in the high-risk score group compared to the low-risk score group (Figure 8G and Supplementary Table 17).

## Discussion

The tumor microenvironment was comprised of an intricate system of immune cells and stromal cells, which contained promising biomarkers of clinical outcome and novel targets for therapeutic approaches (Garcia-Gomez et al., 2018). However, the therapeutic responses and prognosis of non-cancer cells were still poorly understood in OC. The abundance of macrophages and CD8^+^ T cells in the tumor microenvironment were significantly associated with prognosis and immunotherapy response in various cancers (He et al., 2019). A large number of studies have demonstrated that the presence of tumor-infiltrating lymphocytes was associated with a favorable prognosis in OC patients (Santoiemma and Powell, 2015). Anti-immune checkpoint therapy, including PD-1, PDL-1, and CTLA-4, significantly prolonged the survival time of patients with solid tumors (Topalian et al., 2016). Unfortunately, it was a difficult and laborious thing to quantify tumor-infiltrating immune cell subsets with histological inspection in a large patient cohort. The previous studies were limited to only one or two immune cell types, so the results did not reflect overall characterizations of tumor microenvironment cell infiltration due to various immune cell types (Mami-Chouaib et al., 2018). Recently, next-generation sequencing was used to identify key genetic or epigenetic changes in OC, and computational methods were also developed to estimate the abundances of immune cells and stromal cells based on the immunogenomic profiling (Courcelles et al., 2020). Combining genomic data with those novel computational methods provided an opportunity to dissect the characterizations of tumor microenvironment cell infiltration (Ye et al., 2019). In addition, it would be helpful to develop the potential immune-related biomarkers that could help monitor the immunotherapy response or search for new therapeutic targets (Zeng et al., 2019).

This present study provided the overall characterizations of tumor microenvironment cell infiltration in OC and analyzed immune cells, TMB, immune-related pathways, and immune-related genes in OC, combining these with clinical characteristics. Some of the findings in this present study were consistent with the previous studies (Wei et al., 2016; Yuan et al., 2017). In terms of immune cells, this study found an increasing trend of B cells memory in OC patients with grade 3 compared to grade 2, while B cells naïve showed a decreased trend in OC patients with grade 3 compared to grade 2. One study found that a population of interleukin-10(+) B [IL-10(+) B] cells was preferentially enriched in the ascites, which was associated with naive B cell and memory B cell phenotypes. The frequencies of IL-10(+) B cells were negatively correlated with those of interferon gamma-producing [IFN-g (+)] CD8(+) T cells. It demonstrated an additional regulatory mechanism of IL-10(+) B cells in the tumor microenvironment (Wei et al., 2016). The role of tumor-associated macrophages in the tumor microenvironment remains controversial due to two different polarized subsets. This present study found that M1 macrophages had a significantly decreased trend among stages II, III, and IV, while M2 macrophages had a significantly increased trend among stages II, III, and IV. The meta-analysis of nine studies, including 794 patients, found that the higher ratio of M1/M2 macrophages in ovarian cancer tissues was associated with a favorable overall survival (HR = 0.449, 95% CI = 0.283–0.712, *P* = 0.001) (Yuan et al., 2017). The present study also found that eosinophils, neutrophils, and T cells follicular helper were significantly related to OC survival. One comprehensive analysis of publicly available databases containing 13 studies with more than 2,000 patients revealed that neutrophils were associated with poor overall survival (HR = 1.06, 95% CI = 1.00–1.13) and progression-free survival (HR = 1.10, 95% CI = 1.02–1.13) (Liu et al., 2020). Further investigations of quantitative immune cell infiltrations might contribute to therapeutic advances in OC.

In terms of immune-related pathways, DEGs between immunity-high and -low abundance groups of immune cell infiltration were enriched in multiple immune-related pathways according to GO and KEGG analyses; for example, PD-L1 expression and PD-1 checkpoint pathway in cancer, antigen processing and presentation, Fc gamma R-mediated phagocytosis, T-cell receptor signaling pathway, and natural killer cell-mediated cytotoxicity. The multiple enriched pathways were closely related to tumorigenesis and progress in OC, and some studies tried to use PD-1/PD-L1 pathway inhibitors in cancer. It was reported that the PD-1 receptor and its ligand (PD-L1) were over-expressed in tumor cells and immunology system cells in OC patients. It is possible in the future to apply PD-1/PD-L1 pathway inhibitors in the treatment of ovarian cancer (Piêtak et al., 2018). The previous study also showed that OC cell lines exhibited lower expression of transcripts involved in antigen processing and presentation to immune cells compared to normal tissues. In addition, treatment with clinically relevant low doses of DNMT inhibitors (that remove DNA methylation) increased expressions of antigen processing and presentation and Cancer Testis Antigens in these cells. It indicated that the increase of antigens and antigen presentation might be one mechanism to sensitize patients to immune therapies (Siebenkäs et al., 2017). Due to the important role of T cells in the immune surveillance of OC, we paid increased attention to adoptive T-cell therapies as an immunotherapeutic approach for OC. Chimeric antigen receptors, constructed by incorporating the single-chain Fv fragment to a T-cell signaling domain, such as CD3 æ or the Fc receptor ã chain, endowed T cells with non-major histocompatibility complex-restricted specificity. It suggested that chimeric antigen receptor therapy might be possible to translate from basic research into clinical care of OC (Zhang et al., 2017). Tumor cells pretreated with anti-epidermal growth factor receptor inhibitors showed the increased sensitivity toward NK cell-mediated antibody-dependent cellular cytotoxicity. These data implicated that combination therapies with targeted drug and immune agents would be an effective therapy (Mallmann-Gottschalk et al., 2019). Further investigations of the immune-related pathways might contribute to therapeutic advances in OC.

In terms of mutation gene distribution and immune-related genes in OC, the top 30 gene mutations were plotted, including TP53, TNN, MUC16, CSMD3, NF1, TOP2A, USH2A, HMCN1, RYR2, FAT3, MUC17, LRP1B, APOB, BRCA1, FLG, MACF1, CDK12, DNAH3, RB1, AHNAK, COL6A3, KMT2C, LRP2, LRRK2, SYNE1, MDN1, MYH4, SYNE2, TENM1, and DST. This present study also identified the five-immune-related-gene-signature prognostic model (CCL18, CXCL13, HLA-DOB, HLA-DPB2, and TNFRSF17) with lasso regression, and such model-based high-risk and low-risk groups were significantly related to OC overall survival. Mutations in the tumor genome can cause tumors to express mutant proteins that are tumor-specific and not expressed on normal cells (neoantigens). These neoantigens are an attractive immune target because their selective expression in tumors may minimize immune tolerance as well as the risk of autoimmunity (Yarchoan et al., 2017). This present study found that immune-related score was positively correlated with TMB, indicating that identification and targeting of tumor neoantigens would help cancer immunotherapy. Some high mutation genes in OC were also identified, including TP53, TNN, MUC16, CSMD3, NF1, TOP2A, USH2A, HMCN1, RYR2, FAT3, MUC17, LRP1B, APOB, BRCA1, FLG, MACF1, CDK12, DNAH3, RB1, AHNAK, COL6A3, KMT2C, LRP2, LRRK2, SYNE1, MDN1, MYH4, SYNE2, TENM1, and DST. For example, MUC16 inhibited cytolysis *via* human NK cells as well as the formation of NK-tumor conjugates. Mice implanted with MUC16-knockdown OVCAR-3 showed a more than twofold increase in terms of survival compared to controls (Felder et al., 2019). CCL18, one of the cytokines, displayed chemotactic activity for naive T cells, CD4^+^ and CD8^+^ T cells, and non-activated lymphocytes but not for monocytes or granulocytes. The overexpression and silencing of CCL18 affected adhesion, invasion, and migration in OC cells, which suggested that CCL18 has potential as a clinical marker for early diagnosis of OC and as a target molecule in the treatment of OC (Yang et al., 2019). Further investigations of the mutation gene distribution and immune-related genes may contribute to therapeutic advances of OC. Furthermore, the associations of top mutation gene expression with drug sensibility were obtained, and those mutation genes were potential targets. Some drugs were significantly related to OC mutation genes, including drugs dasatinib, zoledronate, epothilone B, pelitrexol, vemurafenib, dabrafenib, PD-98059, tamoxifen, and nelarabine. Some drugs have proven to be effective in OC (Chou et al., 2005; Xiao et al., 2015; Hou et al., 2017; Woo et al., 2017). For example, dasatinib, as a tyrosine kinase inhibitor, has been tested for its antitumor activity in six ovarian cancer cell lines with and without combination with paclitaxel. Its combination with paclitaxel or dasatinib alone showed anti-ovarian cancer properties by inducing cancer cell apoptosis (Xiao et al., 2015). A case of metastases from gastric cancer to the ovary reported that alkaline phosphatase levels remarkably decreased after treatment with zoledronic acid. According to follow-up data of the patient treated with S-1 plus oxaliplatin and zoledronic acid, the cancer status continued to remain in good condition (Woo et al., 2017). One study developed a new class of epothilones, 26-trifluoro-(E)-9,10-dehydro-12,13-desoxy-epothilone B (Fludelone). Results *in vitro* experiment indicated that Fludelone can be used against SK-OV-3 (ovary) xenograft tumors, which indicated that Fludelone could be a promising compound for cancer chemotherapy in OC (Chou et al., 2005). The cisplatin-resistant ovarian cancer cell line (SKOV-3/DDP) exhibited increased resistance *via* the increased phosphorylation of ERK and the enhanced epithelial mesenchymal transition (EMT) process. The specific ERK inhibitor PD-98059 efficiently impaired the cisplatin-resistance of ovarian cancer cells and decreased cell proliferation and migratory area by inhibiting the ERK pathway and EMT process (Hou et al., 2017). Those drug sensibility-associated mutation genes were the potential drug therapeutic targets in OC.

For effective evaluation of the prognosis of OC patients, lasso regression identified the five-immune-related gene signature model (CCL18, CXCL13, HLA-DOB, HLA-DPB2, and TNFRSF17). The risk score derived from this prognostic signature model could be one of the risk factors for OC alongside age at initial pathological diagnosis, anatomic neoplasm subdivision, cancer status, primary therapy outcome, and residual tumor disease. Those five immune-related genes in the prognostic signature model played important roles in immune and cancer (Gu-Trantien et al., 2017; Chenivesse and Tsicopoulos, 2018). For example, CCL18, as one of the Cys-Cys (CC) cytokine genes, displayed chemotactic activity for non-activated lymphocytes, CD4^+^ T cells, naive T cells, and CD8+ T cells but not for granulocytes and monocytes. It might play a role in both humoral and cell-mediated immunity responses. Finally, CCL18 was involved in immune tolerance toward cancer to influence cancer cell activation and migration (Chenivesse and Tsicopoulos, 2018). CXCL13, as a B lymphocyte chemoattractant, has been widely implicated in the pathogenesis of inflammatory conditions and autoimmune diseases, which preferentially promoted the migration and chemotaxis of B lymphocytes by stimulating calcium influx (Kazanietz et al., 2019). Recent studies proved that CXCL13 could control the cancer cell phenotype in various solid tumors and impact the migration, invasiveness, and growth of cancer cells (Gu-Trantien et al., 2017). This generic method had been used in other kinds of cancers, including lung cancer (Wu et al., 2020), invasive ductal carcinoma (Bao et al., 2019), and colon cancer (Wang et al., 2020). Comparative analysis of all these constructed immune-related gene signature models indicated that different cancers had their own individual prognostic models. Thus, it was necessary to analyze the immune microenvironment in different cancers. This was the first time OC data were comprehensively analyzed on TCGA with this generic method to construct five-immune-related gene signature model (CCL18, CXCL13, HLA-DOB, HLA-DPB2, and TNFRSF17). Dissecting the molecular and signaling events of immune-related genes in OC and how those identified genes dynamically control the interaction between cancer cell and tumor microenvironment might be crucial to identify novel effectors and therapeutic targets.

### Scientifical Significance of the Related Findings

Scientifical significance is different from statistical significance in terms of the concept aspect, though both have a certain overlapping (Zhan et al., 2017). When we got a statistically significant finding, we must rationalize it in a biological system from an angle of scientific significance. For this discovery study based on large-scale omics data derived from ovarian cancers, in order to guarantee the scientific significance of the related findings in current manuscript, we assure that each statistical result must be statistically significant with at least *p* < 0.05; then, these statistically significant results were rationalized in the biological system with a scientific explanation. For example, the five-immune-related-gene prognostic signature model (CCL18, CXCL13, HLA-DOB, HLA-DPB2, and TNFRSF17) constructed with lasso regression based on the TCGA ovarian cancer database was verified with a GEO ovarian cancer dataset, and these genes were scientifically rationalized in the biological system of ovarian cancers as described in the section of discussion. Another example, for the mutation study, is that the top mutation gene expressions of OC were statistically significantly associated with drug sensibility, and those mutation genes were thought to be potential targets in OC, such as the drugs dasatinib, zoledronate, epothilone B, pelitrexol, vemurafenib, dabrafenib, PD-98059, tamoxifen, and nelarabine. Some of these drugs have been proven to be effective in OC, as described in the discussion. It clearly demonstrates that the statistically significantly related findings in the current study are also scientifically significant, which is of important scientifical merit in the treatment of OC patients.

## Conclusion

Tumor microenvironment alterations participate in carcinogenesis and development in OCs. Recent studies have revealed that mutation gene distribution and immune-related genes play critical roles in regulating the reprogramming of immune status in cancer cells. In this study, ssGSEA was a useful tool to quantify the relative abundance of immune cell infiltration and recognize immune subtypes. Systematical analysis of the relationships between immune subtype, clinical characteristics, ESTIMATE results, and mutation information could provide new insights into the clinical outcome prediction of OC. Lasso regression identified the five-immune-related gene signature model (CCL18, CXCL13, HLA-DOB, HLA-DPB2, and TNFRSF17) to improve the prediction accuracy for overall survival in OC. Further quantitative investigations of cellular immune infiltrations in tumors may contribute to therapeutic advances. Identification of combination roles of multiple key molecules in recognition of heterogeneity and complexity of tumor microenvironment cell infiltration will reveal the potential mechanisms of tumor microenvironment antitumor immune responses and determine more effective immunotherapy strategies.

## Data Availability Statement

The original contributions presented in the study are included in the article/Supplementary Material, further inquiries can be directed to the corresponding author/s.

## Author Contributions

NL designed the project, analyzed the data, prepared the figures and tables, and wrote the manuscript. BL participated in partial bioinformatics analysis. XZ conceived the concept, supervised the results, critically revised and wrote the manuscript, and was responsible for its financial supports and the corresponding works. All authors approved the final manuscript.

## Conflict of Interest

The authors declare that the research was conducted in the absence of any commercial or financial relationships that could be construed as a potential conflict of interest.

## Footnotes

1 https://portal.gdc.cancer.gov/

2 http://www.bioconductor.org/packages/release/bioc/html/GSVA.html

3 https://www.rdocumentation.org/packages/sparcl/versions/1.0.3

4 https://r-forge.r-project.org

5 http://cibersort.stanford.edu/

6 https://bioconductor.org/packages/release/bioc/vignettes/maftools/inst/doc/maftools.html

7 https://discover.nci.nih.gov/cellminer/

8 https://www.immport.org/home

9 https://www.rdocumentation.org/packages/clusterProfiler/versions/3.0.4

10 https://www.gsea-msigdb.org/gsea/msigdb/index.jsp

11 https://string-db.org/

12 https://cran.r-project.org/web/packages/glmnet/index.html

13 https://www.ncbi.nlm.nih.gov/geo/query/acc.cgi?acc=GSE13876
